# Rapid Determination of Different Ripening Stages of Occidental Pears (*Pyrus communis* L.) by Volatile Organic Compounds Using Proton-Transfer-Reaction Mass Spectrometry (PTR-MS)

**DOI:** 10.3390/foods13040620

**Published:** 2024-02-19

**Authors:** Yuanmo Wang, Qingzhen Zhu, Songzhong Liu, Leizi Jiao, Daming Dong

**Affiliations:** 1 School of Agricultural Engineering, Jiangsu University, Zhenjiang 212013, China; wangym@njau.edu.cn (Y.W.); qingzhen_zhu@ujs.edu.cn (Q.Z.); dongdm@nercita.org.cn (D.D.); 2 Research Center of Intelligent Equipment, Beijing Academy of Agriculture and Forestry Sciences, Beijing 100097, China; 3 Institute of Forestry & Pomology, Beijing Academy of Agriculture and Forestry Sciences, Beijing 100097, China; szliu1978@163.com

**Keywords:** volatile organic compounds, proton-transfer-reaction mass spectrometry, Occidental pear, fruit ripening, heatmap clustering, principal component analysis

## Abstract

Determination of Occidental pear (*Pyrus communis*) ripening is difficult because the appearance of Occidental pears does not change significantly during the ripening process. Occidental pears at different ripening stages release different volatile organic compounds (VOCs), which can be used to determine fruit ripeness non-destructively and rapidly. In this study, VOCs were detected using proton-transfer-reaction mass spectrometry (PTR-MS). Notably, data were acquired within 1 min. Occidental pears harvested at five separate times were divided into three ripening stages: unripe, ripe, and overripe. The results showed that the composition of VOCs differed depending on the ripening stage. In particular, the concentrations of esters and terpenes significantly increased during the overripe stage. Three ripening stages were clearly discriminated by heatmap clustering and principal component analysis (PCA). This study provided a rapid and non-destructive method to evaluate the ripening stages of Occidental pears. The result can help fruit farmers to decide the optimum harvest time and hence reduce their economic losses.

## 1. Introduction

Occidental pears (*Pyrus communis* L.), a member of the family *Rosaceae*, have been cultivated in Europe since 1000 BCE. Occidental pears were introduced into China around 1890 and are now widely cultivated in Shandong, Beijing, Henan, Liaoning, and other provinces [1]. Occidental pears are rich in nutrients, containing vitamins A, B_2_, and C as well as potassium, calcium, iron, and other essential elements of the human body. In addition, they contain fructose and glucose, which can promote digestion and are beneficial to human health. Occidental pears are harvested from August to September. They are unsuitable for eating at the time of harvest and usually require post-harvest ripening before they are placed on shelves. The time of picking is strict, because picking too early or too late will affect the taste and hence consumers’ purchase decisions. Moreover, if they are picked too late, then the pears become overripe during transportation, leading to economic losses. Therefore, it is important to determine the ripeness of Occidental pears accurately. 

Traditionally, the ripeness of fruits is judged by experienced fruit farmers, which is a very subjective assessment. Alternatively, instruments are used to determine parameters related to ripeness, such as the firmness and soluble solid content (SSC), which inevitably destroys the fruit [2]. Recently, many non-destructive detection techniques have been developed to evaluate fruit ripeness. For example, automatic grading systems based on machine vision provide a lot of convenience for the judgment of fruit ripeness, and the recognition accuracy rates can reach 98% after artificial neural networks (ANNs) are integrated [3,4,5]. However, this system may be challenged when the appearance of the fruit does not change significantly during the ripening period. In contrast, spectroscopy-based methods, such as near-infrared (NIR) spectroscopy and hyperspectral imaging, provide characteristic band information to construct models for assessing fruit ripeness. Khodabakhshian and Emadi employed a hyperspectral imaging system to assess the ripeness of pears within the visible and short near-infrared regions (425–1000 nm). The classification of ripening stages was conducted through Partial Least Squares–Discriminant Analysis (PLS-DA), Soft Independent Modeling of Class Analogy (SIMCA), and Linear Discriminant Analysis (LDA). Notably, the optimal correct classification rate achieved was 87.86%, attributable to the application of PLS-DA [6]. Researchers also reviewed some of the latest work in fruit quality assessment using NIR spectroscopy, both from device and model aspects [7,8,9]. However, there are other problems, such as complex data processing and poor universality. Other non-destructive methods for assessing fruit ripeness include dielectric spectroscopy and acoustic vibration techniques, although the results fall short of application standards [10,11].

Like people, fruits also breathe and release volatile organic compounds (VOCs) that are characteristic of their state. For example, during spoilage, fruits can release ethanol and a series aldehydes and terpenes [12]. VOCs released during different ripening stages also have many differences [13]. Additionally, VOCs are an important component of fruit aroma and hence will greatly influence consumers’ purchasing decisions. Gas chromatography–mass spectrometry (GC–MS) is the gold standard of gas detection methods. Many researchers have used GC–MS to investigate fruit ripening, providing reliable information on the changes in VOCs during fruit ripening [14,15]. However, GC–MS relies heavily on laboratory equipment and requires complex sample preparation and lengthy chromatographic separation, which makes rapid, on-site measurements impossible. Compared with GC–MS, spectroscopy-based methods, such as infrared and laser spectroscopy, are fast, do not require sample pretreatment, and can be used to analyze multiple components simultaneously [16]. For example, Fourier-transform infrared (FTIR) spectroscopy and molecular sieves were used to evaluate the ripeness of mango and identify three ripening stages. Peaks at 2080–2045 cm^−1^, 797–787 cm^−1^, and 760–700 cm^−1^ were consistently observed throughout the entire maturity period, with their intensities exhibiting a gradual increase during storage. Remarkably, accurate classification rates of 100% were achieved for the mature green stage, the half-ripened stage, and the fully ripened stage [17]. Zhou et al. innovatively devised a rapid, in situ method employing fiber-optic evanescent wave (FOEW) spectroscopy for the detection of volatile compounds in grapes. This approach successfully and precisely classified three distinct spoilage stages [18]. However, spectroscopy methods have challenges that need to be overcome, such as a high limit of detection and overlapping spectral peaks. The electronic nose (E-nose) is a gas-sensing device consisting of an array of sensors. Owing to its low cost and portability, the E-nose has been rapidly developed in recent years [19,20]. For instance, a non-destructive system for fruit ripeness monitoring was proposed by integrating an E-nose and a camera. This innovative method adeptly identifies the four ripening stages of bananas [21]. Furthermore, comprehensive reviews on E-noses have been published. In general, the E-nose exhibits promising prospects not only in fruit ripeness detection but also in the quality inspection of food products [22,23]. However, its cross-sensitivity and poor repeatability restrict its practical application. 

Unlike the above techniques, proton-transfer-reaction mass spectrometry (PTR-MS) enables on-site, real-time measurements with a limit of detection reaching the parts-per-billion (ppb) level. It has become a powerful tool for the detection of food VOCs. PTR-MS arises from the idea of chemical ionization, and it combines the drift tube model and flowing afterglow technique of the last century. Lindinger, an Austrian scholar, first developed PTR-MS and applied it to the detection of food VOCs [24]. The proton-transfer reaction between VOCs and H_3_O^+^ generated by the PTR-MS ion source occurs in the drift tube. Then, the protonated VOCs are captured by the mass spectrometer. Because the proton affinity of H_2_O is below that of most VOCs but above that of the main components of air (N_2_, O_2_, CO_2_), H_3_O^+^ will only react with VOCs. This allows PTR-MS to use air as the carrier gas, which greatly reduces the size of the equipment. PTR-MS has been applied to diverse domains for detecting VOCs in the context of food analysis. Rozanska et al. employed Proton-Transfer-Reaction Time-of-Flight Mass Spectrometry (PTR-TOF-MS) to discriminate between three distinct citrus fruit species. The primary VOCs in citrus were conclusively identified, leading to the clear separation of these species [25]. Evaluating the potential of PTR-TOF-MS in monitoring the evolution of volatile compounds during post-harvest ripening, the researchers analyzed the compounds released from three apple cultivars [26]. Analogous studies have been conducted on various fruits, including strawberries [27] and tropical fruits (avocado, banana, mango, and mangosteen) [28]. By analyzing the VOC profiles of fruits, researchers gain insights not only into the alterations in VOCs during the ripening process but they can also select VOCs preferred by consumers. Consequently, these identified compounds can be targeted for the cultivation of high-quality genotypes. Farneti et al. comprehensively characterized raspberry VOCs across different ripening stages (pink, ripe, and overripe). The results facilitated the discrimination of the best-performing genotype based on VOCs, providing a valuable candidate for future breeding programs [29]. Overall, PTR-MS emerges as an innovative tool for the analysis of VOCs in fruits [30]. Majchrzak et al. have reviewed some applications and key problems of PTR-MS in the detection of food VOCs [31]. However, to the best of our knowledge, PTR-MS has not been used to determine the ripening stages of Occidental pears. 

In this study, PTR-MS was used to analyze the VOCs of Occidental pears at different ripening stages. The objectives of this study were to rapidly determine the ripening stages of Occidental pears from their released VOCs and to verify the potential of PTR-MS as a rapid gas analysis tool. Three ripening stages were clearly identified. The results provided information for determining the optimal harvest time of Occidental pears, which could be used to reduce the economic losses of fruit farmers. 

## 2. Materials and Methods

### 2.1. Plant Materials

Fresh Occidental pears (*Pyrus communis* ‘Santa Maria’) were picked at the Institute of Forestry and Pomology, Beijing Academy of Agriculture and Forestry Sciences (Haidian District, Beijing, China). Fruits with similar shapes and no scars on their surfaces were collected five times from 29th July to 25th August. Six to eight fruits were picked every seven days. The resulting five batches of samples were named sequentially PA, PB, PC, PD, PE. According to the growth period, they were divided into three ripening stages: unripe (PA and PB), ripe (PC and PD), and overripe (PE). 

### 2.2. Physical Parameters Assessment 

A fruit penetrometer equipped with a 4 mm diameter probe was used to determine the fruit firmness (N). Each sample was measured at three different positions in the equatorial region with a penetration depth of 10 mm. The maximum force was recorded automatically by the penetrometer. 

The soluble solid content (SSC, %) was measured using a hand-held refractometer. The juice was squeezed from three different slices of each pear and dropped on the prism surface of the refractometer, and the SSC was read in the eyepiece. The refractometer was calibrated using distilled water at room temperature (25 ± 1 °C) before each measurement. 

### 2.3. VOCs Measurement 

**Figure 1:**
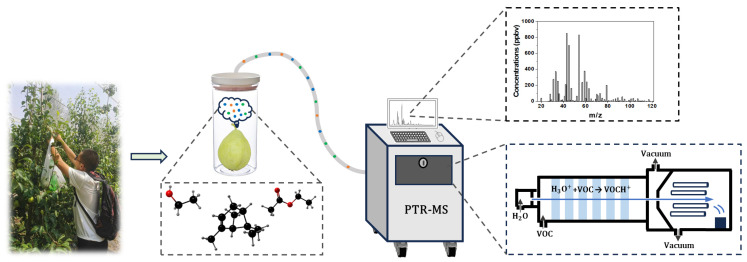
The flow chart of Occidental pear VOCs detection based on PTR-MS.

The harvested Occidental pears were taken to the Research Center of Intelligent Equipment, Beijing Academy of Agriculture and Forestry Sciences (Haidian District, Beijing, China) in 30 min for further analysis. The whole sampling process was finished in 5 min. During transportation, each pear sample was stored in a sealed bag to maintain its status and avoid interference among the pears. The workflow is shown in Figure 1. 

The PTR-MS system (YWHJ-MP-510, Hefei Institutes of Physical Science, Chinese Academy of Science, Hefei, Anhui province, China) used in this study was equipped with a quadrupole mass spectrometer, which can separate ions by their mass. The proton donor, hydrated hydrogen ions (H_3_O^+^), was generated by a hollow cathode. Scanning was conducted in the range of mass-to-charge ratio (m/z) 17–121, and the scanning time of each m/z was 100 ms. The analyte gases were measured via a heated (80 °C) inlet tube with a flow rate of 500 ± 100 standard cubic centimeters per minute (sccm). 

Each pear was placed in a glass jar with a volume of 1 L for 30 min to accumulate VOCs. An empty jar was set up to collect air, representing the background, and three jars with samples were set up as the experimental group. The m/z 33 signal was taken as the reference data, and after the m/z 33 signal stabilized (approximately 1 min), the air inlet was inserted into the glass jar for 2 min to measure the full spectrum of the VOCs. Between two measurements, ambient air was injected into the PTR-MS for 1 min to eliminate the effects of the last measurement. To sum up, every single measurement was finished in 5 min (1 min for signal stabilizing, 2 min for the measurement itself, and 1 min for memory effect elimination). Accordingly, the glass jars were also sealed every 5 min to control 30 min accumulation time. All the measurements were taken at room temperature.

### 2.4. Statistical Analysis

The concentrations of VOCs were calculated using CAS PTR DATA 2.4.9 (Hefei Institutes of Physical Science, Chinese Academy of Science, Hefei, China). The concentrations were performed in ppbv. The data were imported to Office Excel 2019 (Microsoft, Redmond, WA, USA) for further processing. The valid data were processed by calculating the maximum value of each m/z signal minus the mean value of the background (air) signal. The concentrations were reported as the average of three replicates, and the firmness and SSC were tested fifteen times. For heatmap clustering, some m/z data (standard deviation below 1) were deleted. The heatmap and other plots were created using Origin 2023b (10.0.5.153, OriginLab, Northampton, MA, USA). Principal component analysis (PCA) was performed using Unscrambler X (10.4, CAMO Software, Oslo, Norway) to discriminate the Occidental pears according to the ripening stage.

## 3. Results and Discussion

### 3.1. Changes in Physical Parameters 

**Figure 2:**
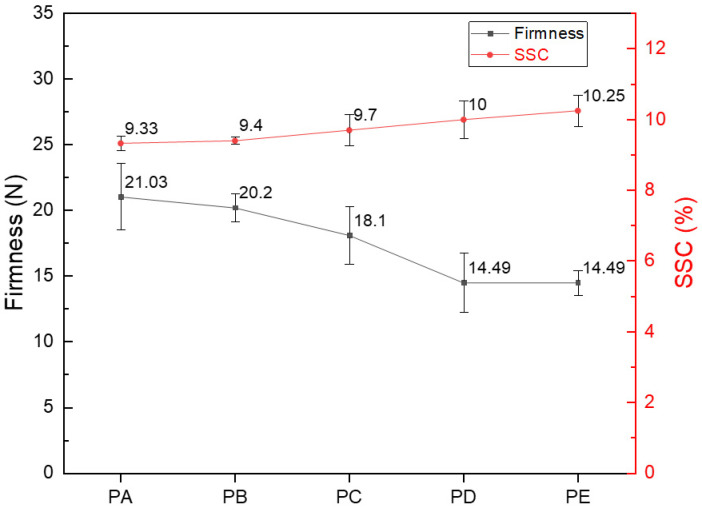
Changes of the firmness and soluble solid content (SSC) of Occidental pears at different ripening stages. Error bars over the line represent the standard deviation ± SD of means (n = 15).

As Occidental pears ripen, some physical parameters start to change, among which firmness and SSC are important indicators of pear ripening [2]. As shown in Figure 2, from PA to PE, the firmness gradually decreased, while the SSC gradually increased. The firmness changed at a greater rate from PB to PC and from PC to PD than from PA to PB, probably because at the time of the first two picks, the Occidental pears were still unripe. After the second pick, ripening accelerated, and thus the firmness decline also accelerated, which was consistent with the results reported by Bourne and Vangdal [32,33]. However, from PD to PE, the firmness remained unchanged, which was attributed to the pears entering the overripe stage. The regularity of the firmness change was consistent with the characteristics of the three ripening stages of the growth period. 

During the ripening period, the starch in the pears is converted into sugar, which increases the SSC. However, the SSC of pears picked at the overripe stage is below the ideal level for eating. The minimum acceptable SSC is 14% [34]. According to fruit farmers, Occidental pears require post-harvest ripening to enhance their flavor. Pears picked at the overripe stage are already vulnerable. If they are picked later, there would be no more time for transportation, and by the time they are placed on shelves, the pears would have rotted, which leads to economic losses for sellers. According to the results of the firmness and SSC measurements, the optimal picking time was at the ripe stage. 

**Figure 3:**
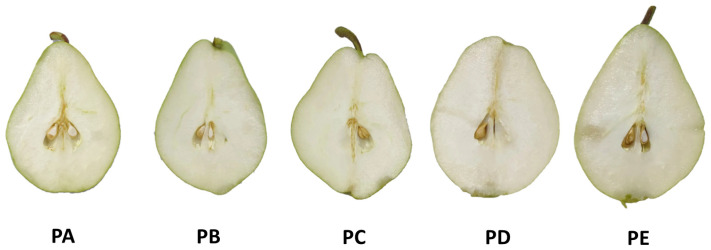
Details of sectioned Occidental pears at different ripening stages: unripe (PA and PB), ripe (PC and PD), and overripe (PE).

From PA to PE, the color of Occidental pears did not change significantly. However, the seed coat gradually darkened in color (Figure 3). Although experienced fruit farmers can judge the ripeness from the color of the seed coat, this method depends on personal experience and damages the pears, which makes it unsuitable for practical application. Nevertheless, a variety of VOCs are emitted by Occidental pears during the ripening period, which can be analyzed to determine the ripening stage [1].

### 3.2. Dynamic Changes of VOCs

**Figure 4:**
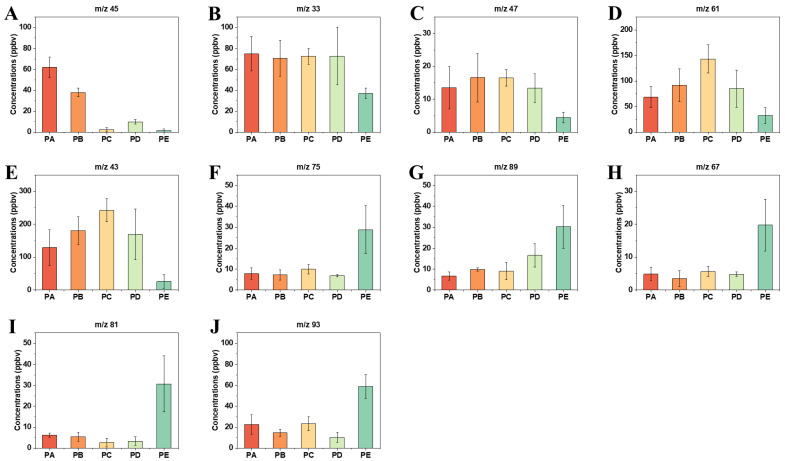
(**A**–**J**) Changes in concentrations (ppbv) of characteristic m/z at different ripening stages. Error bars over the column represent the standard deviation ± SD of means (n = 3).

We analyzed the concentration changes of all the m/z and identified some characteristic m/z with significant concentration changes. Their tentative identifications are shown in Table 1, including alcohols, aldehydes, esters, terpenes, and other VOCs commonly found in fruits. The concentration changes at the five harvest times were different. The concentration of the m/z 45 (acetaldehyde) markedly decreased from 60 ppbv to less than 5 ppbv after entering the ripe stage (Figure 4A). Aldehydes can provide a strong fruit flavor with fruity, fresh, and green leaves [35]. As fruits ripen, the flavor gradually weakens. Chen et al. found that during storage, the concentrations of aldehydes released by all five species of pears decrease by different degrees depending on the species [36]. A significant decrease in aldehydes during the ripening process was also seen in the research of other fruits like hawthorn [37] and feijoa [14]. In addition, we found that the concentrations of alcohols, such as m/z 33 (methanol) and m/z 47 (ethanol), also decreased (Figure 4B,C). However, the concentration decline of alcohols occurred later than that of aldehydes. Alcohols are biosynthesized from unsaturated fatty acids [14]. The decrease in concentrations of fatty acids (see next paragraph) in the overripe period might be the reason for this delay.

A large number of aromas of Occidental pears originate in the VOCs produced through fatty acid-derived pathways. Straight-chain esters, such as methyl acetate and ethyl acetate, are typical fatty acid-derived VOCs [1]. m/z 61 and m/z 43 were identified as acetic acid and its fragment, respectively. They showed a very similar trend of first increase and then decrease. At the ripe and overripe stages, the concentrations of m/z 61 and m/z 43 decreased (Figure 4D,E), probably because they were involved in the formation of esters. Accordingly, the concentrations of m/z 75 (methyl acetate) and m/z 89 (ethyl acetate) increased during this period (Figure 4F,G), which has proven the guess. Researchers investigating apples and peaches have highlighted the contributions of m/z 61 and m/z 43 to esters [39,40]. 

Terpenes, such as monoterpenes and sesquiterpenes, are found in many fruits [28]. Due to possible collision in the drift tube, some fragments are produced during the proton-transfer reaction, which complicates VOC identification. We assigned these fragments by referring to the fragmentation patterns of some monoterpenes reported by Tani et al. [41]. As a result, m/z 67 and m/z 81 were derived from α- and β-pinene, 3-carene, and limonene, whereas m/z 93 was derived from p-cymene. The concentrations of these m/z showed a significant increase in the overripe period (Figure 4 H–J). Monoterpenes and their fragments have been observed in the research on citrus and mangoes [25,42]. Accurate identification of some compounds, especially isomers, is a problem for PTR-MS. Getting the inspiration from selected ion flow tube mass spectrometry (SIFT-MS), researchers have modified PTR-MS with switchable reagent ion (SRI) to overcome this problem [43]. The reactions between isomers and different reagent ions can provide extra information for compound identification. Commonly used switchable reagent ions include NO^+^ and O_2_^+^. For example, Shen et al. used NH_4_^+^ to increase the sensitivity of detection [44]. 

### 3.3. Heatmap Clustering and PCA

**Figure 5:**
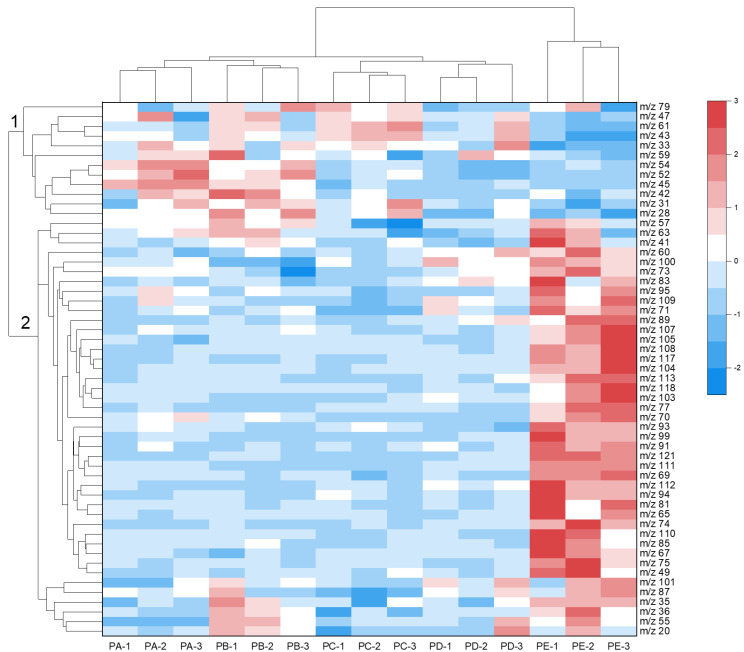
The clustering heatmap of m/z of Occidental pears at different ripening stages. The concentrations of each m/z are shown in different colors, with darker reds representing higher concentrations and darker blues representing lower concentrations. The concentrations of each m/z were normalized. Each m/z is represented by a single row, and each measurement is marked in a single column. The numbers on the left of the tree diagram indicates the groups where m/z belong.

Heatmap clustering, also referred to as hierarchical cluster analysis (HCA), stands out as a valuable multivariate analysis method, particularly adept at handling large datasets. This technique is instrumental in elucidating similarities among distinct data groups and effectively clustering those that share similarities [45]. Heatmap clustering has found widespread application in omics research. Notably, Shi et al. employed liquid chromatography–mass spectrometry (LC-MS) to analyze the nutritional composition of crops and fruits, identifying over 260 nutrients. Through clustering analysis, the nutritional content of each crop and fruit was delineated, providing valuable insights that can guide consumers towards maintaining a healthy diet [46]. To display the changes in VOCs at different ripening stages, we created a clustering heatmap (Figure 5) by grouping the m/z with similar features and centralizing the data distribution. In the heatmap, the darker red represents the higher concentration, while the darker blue represents the lower. The tree diagram at the top of heatmap indicates that the data were well clustered. Among them, PA and PB, representing the unripe stage, were clustered together, while PC and PD, representing the ripe stage, were clustered together. In contrast, PE, representing the overripe stage, was clustered alone owing to its unique characteristics. Therefore, the heatmap clustering of released VOCs can be used to determine the ripening stages of Occidental pears. The tree diagram on the left of the heatmap shows that m/z with significant concentration changes, totaling 55, were clustered in two groups. The first group, located at the top of the heatmap, included m/z 61 (acetic acid), m/z 43 (fragment of acetic acid), m/z 45 (acetaldehyde), m/z 33 (methanol), m/z 47 (ethanol), and other m/z with high concentrations at the unripe and ripe stages. As ripening proceeded, their concentrations gradually decreased. A change in their concentrations indicates a change from the unripe to the ripe stage, which in turn indicates the picking time. For example, a decrease in the concentrations of aldehydes and organic acids indicates the onset of the ripe stage, which is the optimal picking time (Section 3.1). The second group of VOCs included m/z 67 (fragment of terpenes), m/z 75 (methyl acetate), m/z 81 (fragment of terpenes), m/z 89 (ethyl acetate), m/z 93 (fragment of monoterpenes), and other compounds with high concentrations at the overripe stage. They are the main source of the aromas of Occidental pears. However, an increase in their concentrations indicates that the optimal picking time has passed.

**Figure 6:**
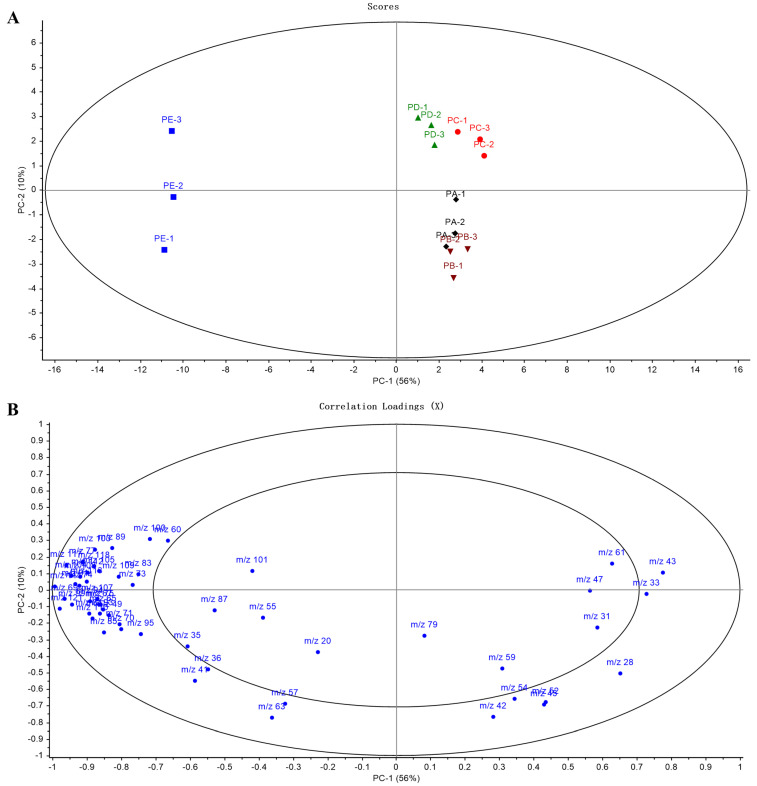
Principal component analysis (PCA) of Occidental pears at different ripening stages. (**A**) Scoring plot showing the contribution of principal components and distribution of pear samples: unripe stage (PA—black diamonds and PB—inverted brown triangles), ripe stage (PC—red circles and PD—green triangles), overripe stage (PE—blue squares). (**B**) Loading plot showing the contribution of each m/z in the measurement of Occidental pears at different ripening stages.

PCA is a commonly used unsupervised dimension reduction method, which can be used to find the most representative variables in the dataset. Figure 6A shows the PCA scoring plot of VOCs detected at different ripening stages. The Occidental pears were separated into three groups, which corresponded to the three ripening stages. The first and second components of the PCA scoring plot account for 56% and 10% of the variation, respectively. In PC 1, there was a clear separation between the overripe stage and the other two stages. This segregation is attributed to a pronounced surge in the concentrations of numerous VOCs during the overripe stage. Along the PC 2 axis, the unripe stage was separated from the ripe stage. Overall, the unripe stage and the ripe stage were closer, and were both far from the overripe stage, indicating that the difference of VOCs concentrations between the first two stages were not obvious and the overripe stage had greater differences from the first two, which was consistent with the result of heatmap clustering. The PCA loading plot (Figure 6B) showed that the positive values of PC 1 were significantly influenced by m/z 61 (acetic acid), m/z 43 (fragment of acetic acid), m/z 45 (acetaldehyde), m/z 33 (methanol), and m/z 47 (ethanol), which all belonged to the first group in the heatmap. In addition, the second group contributed to the negative values of PC 1, which played a major role in the determination of the overripe stage. Therefore, the ripening stages of Occidental pears can be determined by analyzing the VOCs released by the fruits. 

## 4. Conclusions

In summary, a rapid, non-destructive method for determining the ripening stages of Occidental pears was established in this study. The VOCs of Occidental pears at different ripening stages were measured using PTR-MS. The three ripening stages were easily identified by heatmap clustering and PCA. By analyzing the dynamic changes in VOCs, some representative VOCs were found. The changes in their concentrations indicated the onset of each ripening stage, allowing identification of the optimal picking time. This information can coach the picking decision of fruit farmers, and the ripe stage was considered the optimal picking time. 

Since no one has ever used PTR-MS to determine the ripening stages of Occidental pears, the method mentioned in Section 2 still needs attempts at standardization and validation. A weak aspect of PTR-MS is compound identification. GC-MS will be applied in parallel to identify VOCs more accurately in the future. Moreover, we will deploy PTR-MS in the orchard to directly analyze the VOCs released by fruits and determine the different ripening stages. As a powerful analytical tool, PTR-MS has the potential to accomplish this goal.

## Figures and Tables

**Table 1:** Tentative identification of characteristic m/z.

m/z	Tentative Identification ^1^	Reference
33	Methanol	[38,39]
43	Fragment of acetic acid	[40]
45	Acetaldehyde	[13,38,39]
47	Ethanol	[13,29,38]
61	Acetic acid	[25,39]
67	Fragment of terpenes	[25]
75	Methyl acetate	[25,39]
81	Fragment of terpenes	[13,25]
89	Ethyl acetate	[13,25,39]
93	Fragment of monoterpenes	[13,25]

^1^ VOCs tentative identifications were concluded based on the corresponding relationships and fragmentation patterns of previous research.

## Data Availability

The raw data supporting the conclusions of this article will be made available by the authors on request.
